# Causes of Stress Among Poles and How They Cope With Stress During the COVID-19 Pandemic

**DOI:** 10.3389/fpsyt.2022.829918

**Published:** 2022-04-06

**Authors:** Estera Twardowska-Staszek, Krzysztof Biel, Irmina Rostek, Anna Seredyńska

**Affiliations:** Institute of Educational Sciences, Jesuit University Ignatianum in Kraków, Kraków, Poland

**Keywords:** COVID-19, coronavirus pandemic, stress, coping strategies, cross-sectional survey

## Abstract

This study aimed to learn about causes of stress among adult Poles and their ways of dealing with stress during the COVID-19 pandemic. A survey questionnaire was used, as well as two standardized research tools: Endler and Parker’s *Coping Inventory for Stressful Situations* (CISS), and Watson and Clark’s *Positive and Negative Affect Schedule* (PANAS). The research group comprised 595 people, including 80.5% women. They were 18–75 years old. The most important stress factors were concern for one’s health, as well as the current political and economic situation in the country. Most of the participants lean toward avoidance-oriented coping with stress, fewer of them prefer emotion-oriented coping, and the remaining ones focus on task-oriented coping. Task-oriented style is typical of those who are older, married and those who have children. Emotion-oriented coping is more common among women, young people, unmarried people and those without children. Avoidance-oriented style is connected with those who are single, childless, and combining study with work. The most adaptive style of dealing with stress in terms of emotions was task-oriented coping. Psychological support focused on strengthening adaptive strategies of coping with stressful situations is an important task for professionals in the field.

## Introduction

The whole world has been struggling with the destructive effects of COVID-19 for 2 years. Since reaching Poland, 5540162 people have become infected and 109792 have died of diseases caused by the virus (1).

The COVID-19 pandemic has created a new reality, difficult to compare with other stressful events involving large groups of people: natural disasters or international mass conflicts (2). It is a stressor that, as psychiatrists assume, will increase the number of people in need of mental health professionals (3). Most research results confirm that the pandemic has contributed to an increase in the level of stress experienced by people as well as an increase in the number of patients suffering from depression and anxiety (4–6).

The pandemic contributed to the experience of stress in two ways. A review of studies shows that fear of COVID-19 was reported by 18.1–45.2% of the general population (7). In addition to the stressor associated directly with infection, there are also several stressors related indirectly to the pandemic, e.g., the general political and economic situation of the country, access to healthcare, individual economic situation, isolation and the lack of social contacts, or simply a fear of an unknown future. The meaning of subsequent factors varies in different countries (8), and may also be related to such variables as race (e.g., 9) or age (e.g., 10).

As a consequence of the experience of stress in various areas of functioning people struggle with various problems, mainly those related to anxiety disorders. According to the research results, stress and post-traumatic disorders especially refer to people who work in health services and their family members (e.g., 11). Also, the pandemic exerted a negative influence on people who had already been suffering from mental disorders (12). Their mental health worsened due to increased fear, isolation and cognitive overload. Negative consequences of COVID-related stressors were also found in general populations. Research carried out on a group of 2,457 Poles has revealed that 77% of them are afraid of contracting a disease, 44% have generalized anxiety disorder, and 86% have felt stressed and nervous within the previous 14 days (13).

Autumn and winter of 2020 was a difficult time in Poland. On October 22, 2020, the Constitutional Tribunal ruled that an abortion is (in most cases) inconsistent with the constitution (14). In response, mass social protests against the tightening of abortion regulations in Poland began. As a result, thousands of citizens participated in protests, which took place almost every day until the end of January 2021. In response, the government took further repressive measures, declaring that assemblies during the pandemic were illegal, which resulted in numerous arrests and sometimes the use of police force against the demonstrators (15). Parallel to the fight for women’s rights, the fight against the pandemic took place. In December, the number of deaths from COVID-19 exceeded 11,000, and the government introduced a national quarantine. In addition to the closure of schools and universities, gastronomy, cultural facilities, entertainment, sports, and religious institutions, the government introduced a limit of people who can meet at the family table during Christmas and announced the introduction of a curfew (16).

When coping with stress various strategies appear to have differing effects in preventing or supporting psychological symptoms (17). Taking into account the results of studies showing that high resilient copers constitute the smallest group in some populations (18) we assume that the issue of resilience in the context of coping should be considered. The analyses (19) indicate that resilience is based on a “3C” foundation: control, coherence, and connectedness with others. They are the basis for interventions taken in order to minimize the negative effects of stress inducing events.

The objective of the research was to analyze sources of stress among adult Poles and their ways of coping with stress during the COVID-19 pandemic. Moreover, the research aimed at specifying the relationship between coping styles and positive/negative emotions in the context of their adaptiveness. Therefore, three research questions were formulated:

1. What are the main sources of stress in adult Poles during the second wave of the COVID-19 pandemic?
2. What styles of coping with stress are used by adult Poles during the COVID-19 pandemic?
3. Which style/s of coping with stress is/are related to positive and which to negative emotions?

## Materials and Methods

### Participants

A total of 595 people took part in the research. The research participants had to meet the following two criteria: they were to be permanent residents of Poland and aged at least 18. Participation in the research was voluntary and anonymous.

### Procedure

The research was carried out in accordance with all Polish and international ethical standards, and with the consent of Ignatianum University’s Research Ethics Committee. The study was carried out with the use of the snowball sampling method in social media. This was an example of *ex post-facto* cross-sectional research, carried out with the use of online survey questionnaires sent through e-mails and social media. The survey was carried out between December 2020 and January 2021.

### Measures

The research participants were asked to fill in an online questionnaire. Demographic variables were collected with the use of *ad hoc* questions. The analyzed demographic variables included sex, age, marital status, children, education, and employment. Moreover, the participants were asked about their perception of the sources of stress during the pandemic.

The participants’ styles of coping with stress were measured using the *Coping Inventory for Stressful Situations* (CISS) by Endler and Parker (20, 21). The questionnaire includes 48 statements related to various behaviors presented by people who experience stressful situations. The respondent is required to provide the answers on a 5-point Likert scale, declaring the frequency of taking up a given activity in difficult situations (from 1–never, to 5–very often). The results of the questionnaire are presented in the form of three styles of coping with stressful situations: task-oriented coping (TOC), emotion-oriented coping (EOC), and avoidance-oriented coping (AOC). The last of these may take the form of either distraction (D) or social diversion (SD).

In order to measure the participants’ emotions, the researcher used the *Positive and Negative Affect Schedule* (PANAS) by Watson and Clark (22, 23). PANAS consists of 20 items–adjectives which denote positive and negative emotions. The participant specifies the intensity of such feelings with the use of a 5-point scale (from 1–very slightly or not at all, to 5–extremely). What we obtain are the results in two sub-scales: Positive (PA) and Negative Affect (NA).

### Data Analysis

The analysis was carried out using the R programme, version 4.0.3. (24). The comparison of the values of quantitative variables in two groups was made with the use of the Mann-Whitney test. The comparison of the values of quantitative variables in three and more groups was made with the use of the Kruskal–Wallis test. After discovering statistically significant differences, the *post hoc* analysis utilizing Dunn’s test was carried out to identify groups with statistically significant differences. The correlations between quantitative variables were analyzed with the use of semi-partial correlations. The level of significance was established as 0.05 in the analysis.

## Results

### Participants

A total of 595 people living in Poland took part in the research. Most participants were women (80.50%). The age range was from 18 to 75 years of age (*M* = 35.95 years). 20.84% of the people surveyed were under 22; 26.22%–from 23 to 34 years of age; 47.06%–aged 35–60; and 5.88% were over 60. Almost half of the participants were married (49.92%), while 41.34% were single. More than fifty percent of the Poles surveyed declared having children (52.10%). 59.33% participants are university graduates, while 27.90% still study. People with secondary education constituted 10.76% of participants; with vocational education–1.85%; with primary education–0.17%. People who worked constituted 53.78% of participants; 19.16% of participants still studied, while 16.47% studied and worked at the same time. Smaller groups of participants included people who do not work (6.72%), as well as retired employees or pensioners (3.87%).

### Sources of Stress

Table 1 shows that the research participants declare that the most common stressors during the pandemic are those related to health (difficulty accessing treatment of other diseases and the possibility of contracting COVID-19 by the closest family and friends-as well as those connected with the current situation in the country, i.e. the political and economic situation in Poland). Interestingly, during the time when the risk of contracting the virus and falling severely ill was greater, only less than one fifth of the participants perceived getting infected with COVID-19 as a source of stress.

**TABLE 1:** Source of stress during the pandemic according to the people surveyed.

Which of the following situations are the most stressful to you?	*n*	% *
Difficulty accessing treatment of other diseases	338	56.81%
Political situation in Poland	335	56.30%
My family members may get infected with COVID 19	283	47.56%
Economic situation in the country	279	46.89%
Lack of social contacts	275	46.22%
Online learning	209	35.13%
Restrictions	170	28.57%
Lack of respirators and medical staff in hospitals	154	25.88%
No job or risk of losing a job	137	23.03%
My family’s financial problems	120	20.17%
Contracting COVID 19	113	18.99%
Other factors	16	2.69%

*The percentage does not add up to 100, because it was not a multiple choice question.

### Styles of Coping With Stress and Positive and Negative Affect

The research results (Table 2) show that the highest scores were obtained by the respondents in the TOC subscale, then in the EOC subscale and finally in the AOC subscale. However, taking into account the norms developed for the tool in the period preceding the pandemic–most participants apply avoidance-oriented coping (high level demonstrated by 39,50%), fewer of them–emotion-oriented coping (high level–37,48%), and still fewer of them use task-oriented style of coping with stress (high level–32,77%).

**TABLE 2:** Participants’ questionnaire means scale scores.

Variables	M	Sd
**CISS**		
TOC	57,16	8,33
EOC	47,74	11,25
AOC	47,01	8,03
D	21,22	5,11
SD	17,26	3,90
**PANAS**		
PA	26,22	7,22
NA	22,30	7,53

*TOC, task-oriented coping; EOC, emotion-oriented coping; AOC, avoidance-oriented coping; D, distraction; SD, social diversion; PA, positive affect; NA, negative affect.*

Likewise, while the raw scores of PANAS do not indicate the advantage of negative over positive emotions, referencing them to norms shows 38.15% had a low level of positive emotions, 31.60% people had a high level of positive emotions, and 30.25% people had a medium level of positive emotions. In the sub-scale of negative emotions, 60% people revealed a high level, 29.08% people revealed a medium level, and 10.92%–a low level (25).

### Coping Styles and Demographic Variables

Table 3 shows the correlations between the styles of coping with stress and demographic variables. The variables connected with task-oriented coping (TOC) are older age, being married, having children, living in a big city, university education, and employment. Emotion-oriented coping (EOC) is more common among women, younger people, singles, childless people, those with secondary and lower-level education, including those who still go to school, as well as among people who combine study and work. Avoidance-oriented coping (AOC) is related to being single, having no children and combining study with employment. Distraction is typical of younger people, singles and people without children. Social diversion is the most common among people with a university degree, as well as those who study and work at the same time.

**TABLE 3:** Task-oriented, emotion-oriented, and avoidance-oriented coping and demographic variables.

Demographic Variables		CISS									
		TOC		EOC		AOC		D		SD	
		Me quartiles	*p*	Me quartiles	*p*	Me quartiles	*p*	Me quartiles	*p*	Me quartiles	*p*
Sex	Women (*N* = 479)	57 51–62.5	*p* = 0.13	49 41–57	*p* < 0.001*	47 42–52	*p* = 0.803	21 18–24	*p* = 0.71	18 15–20	*p* = 0.09
	Men (*N* = 116)	59 51.75–63		44 34–51		46.5 43–52		22 18–24.25		17 14–19	
Age	Under 22–A (*N* = 124)	54 49–59	*p* < 0.001* D,C > B > A	56 47–62.25	*p* < 0.001* A > B > C,D	48 42.75–55	*p* = 0.054	22.5 18.75–25	*p* = 0.023* A > C	17 14–20	*p* = 0.247
	23–34 years–B (*N* = 156)	56 51–62		50 42–57		48 43–54		22 18–25		18 16–20	
	35–60 years–C (*N* = 280)	60 54–64		45 38–51		46 41–51		21 17–24		17 15–20	
	Over 60 years–D (*N* = 35)	59 56–65		43 35.5–47		46 44–51		22 18–24.5		18 15–19	
Marital Status	Single–A (*N* = 259)	56 50–61	*p* < 0,001* B > A	52 44–59	*p* < 0.001* A > B,C	48 42.5–55	*p* = 0.016* A > B,C	22 18–25	*p* = 0.025* A > B,C	17 15–20	*p* = 0.288
	Married–B (*N* = 297)	59 53–65		45 38–52		46 41–51		21 18–24		17 15–20	
	Others–C (*N* = 39)	57 56.5–60		43 36–51.5		46 42–49.5		22 16–23.5		17 14–18.5	
Children	No (*N* = 285)	56 50–60	*p* < 0.001*	52 43–59	*p* < 0.001*	48 43–54	*p* = 0.025*	22 18–25	*p* = 0.005*	17 15–20	*p* = 0.934
	Yes (*N* = 310)	60 53–65		44 38–51		46 41–51		21 17–24		17 14.25–20	
Education	Higher–A (*N* = 353)	60 54–64	*p* < 0.001* A > B,C	46 38–52	*p* < 0.001* C > A,B	47 42–51	*p* = 0.394	21 17–24	*p* = 0.001* B,C > A	18 15–20	*p* = 0.002* A > C,B
	Secondary–B (*N* = 64)	56 48.75–60.25		46 34.75–53.25		46 44–52		23.5 18–26		17 14–19	
	Other–C (*N* = 178)	54 49–59		55 45.25–61		48 42–53		22 18–25		17 14–20	
Employment	Student–A (*N* = 114)	54 49–59	*p* < 0.001* B > C,D,A	55.5 46–61	*p* < 0.001* A,D > C > B	47 39.25–53	*p* = 0.016* D > B,A	21.5 18–25	*p* = 0.059	17 13–19	*p* = 0.005* D > C,A B > A
	Employed–B (*N* = 320)	60 54–64		44 37–51		46 42–51		21 17.75–24		18 15–20	
	Not employed–C (*N* = 63)	68 49.5–61		47 40–54		47 42–51		21 18–25		17 14.5–19.5	
	Employed student–D (*N* = 98)	55 50–60.75		54 47–59.75		49 45–55		22 19.25–25		18 16–20	

*Statistically significant relationship (p < 0.05), TOC, task-oriented coping; EOC, emotion-oriented coping; AOC, avoidance-oriented coping; D, distraction; SD, social diversion.

### Coping Styles and Emotions

Table 4 refers to the relationship between a coping style and positive/negative emotions. There is a positive correlation between TOC and positive emotions. EOC correlates positively with negative emotions and negatively with positive emotions. There are no statistically significant relationships between AOC and emotions.

**TABLE 4:** Semi-partial correlations between CISS and PANAS.

PANAS	TOC	EOC	AOC	D	SD
PA	0,292, *p* < 0,001*	−0,41, *p* < 0,001*	0,032, *p* = 0,433	−0,041, *p* = 0,321	0,044, *p* = 0,283
NA	0,045, *p* = 0,276	0,517, *p* < 0,001*	−0,026, *p* = 0,526	0,023, *p* = 0,578	0,005, *p* = 0,897

*Statistically significant relationship (p < 0.05), TOC, task-oriented coping; EOC, emotion-oriented coping; AOC, avoidance-oriented coping; D, distraction; SD, social diversion; PA, positive affect; NA, negative affect.

## Discussion

The research shows some specific features of the way in which Poles have experienced the pandemic. For most of the research participants, the threat of contracting SARS-CoV-2 is not the greatest source of stress. What they fear the most is the fact that treatment of other diseases is less available during the pandemic and that the health of their closest family members may be affected. The pandemic has clearly shown that the Polish health service is ill-equipped to deal with the direct and indirect consequences of a health crisis. The results of our research confirm that the country’s political and economic situation is a significant stressor for Poles. The feeling of being betrayed and abandoned by state institutions correlates with negative emotions (26).

The research results seem to correlate with the data that suggest the adaptive importance of “3C” (control, coherence, and connection) in coping with pandemic stress (27).

Task-oriented coping is related to controlling the surrounding reality and re-formulating the assessment of the situation from threat into challenge. In the context of the pandemic, it may be reflected in taking up tasks reducing the threat of contracting the virus, as well as planning everyday activities, searching for reliable information about the virus, etc. This style of coping has a positive correlation with positive emotions. Complementary results were obtained by Italian researchers who concluded that a sense of self-effectiveness and focusing on a problem strengthen our ability to manage negative emotions (28). This coping style is typical of older people, people who are married, people with children, and employed people, all of which are connected with a more stable lifestyle and responsibility for others.

Coherence, which provides meaning to what is happening, relates to recognizing, naming and accepting emotions that accompany difficult events. Emotion-oriented coping, the essence of which is focusing on one’s own feelings, yet combined with taking up actions that aim at releasing emotional tension, seems to be a non-adaptive solution as it negatively correlates with positive emotions and has a positive correlation with negative emotions Similar conclusion were brought by the research indicating a strong correlation between emotional style and depression (17). This coping style is more frequent among younger people, people without children and those with lower levels of education.

Many studies show that connecting with others, remaining in meaningful relationships, perceived social support, has a positive effect on psychological wellbeing (29). At first, analyzing simple correlations between emotions and styles of coping, we found a relationship between social diversion and positive emotions (*r* = 0.26; *p* < 0.001). However, more advanced analyzes did not confirm the existence of such a relationship. Thus, although immersion into the world of social relations may have a salutary effect on psychological wellbeing, several studies (30) show that this effect may be quite opposite. The ambiguity of the obtained results prompts to conduct further research.

The recommendations formulated by the Polish Psychiatric Association (31) indicate the need to pay attention to groups particularly vulnerable to the negative consequences of a pandemic experience: people with pre-pandemic mental disorders history, but also elderly and very young people who do not have enough resources to cope with completely new challenges. Adaptive styles of coping with stress seem to be one of the most important resources in this context. An important task for educators and mental health professionals is to promote and strengthen their use. It may contribute not only to the improvement of the functioning of individuals, but also to the economic recovery of countries (32).

## Data Availability Statement

The raw data supporting the conclusions of this article will be made available by the authors, without undue reservation.

## Ethics Statement

The studies involving human participants were reviewed and approved by The Ethics Committee of Jesuit University Ignatianum in Kraków. The patients/participants provided their written informed consent to participate in this study.

## Author Contributions

ET-S contributed to all the phases of the study, conception and design of the study, results interpretation, and writing and editing of the manuscript. IR contributed to results interpretation, and writing and editing of the manuscript. KB and AS contributed to writing and editing of the manuscript. All authors contributed to manuscript revision, read, and approved the submitted version.

## Conflict of Interest

The authors declare that the research was conducted in the absence of any commercial or financial relationships that could be construed as a potential conflict of interest.

## Publisher’s Note

All claims expressed in this article are solely those of the authors and do not necessarily represent those of their affiliated organizations, or those of the publisher, the editors and the reviewers. Any product that may be evaluated in this article, or claim that may be made by its manufacturer, is not guaranteed or endorsed by the publisher.
